# Brain Activity in Different Brain Areas of Patients With Dry Eye During the Female Climacteric Period According to Voxel-Based Morphometry

**DOI:** 10.3389/fneur.2022.879444

**Published:** 2022-05-24

**Authors:** Meng-Yan Hu, Li-Juan Zhang, Min Kang, Yi-Cong Pan, Qian-min Ge, Qiu-yu Li, Lin Yang, Chong-Gang Pei, Yi Shao

**Affiliations:** Department of Ophthalmology, The First Affiliated Hospital of Nanchang University, Jiangxi Province Ocular Disease Clinical Research Center, Nanchang, China

**Keywords:** voxel-based morphometry, magnetic resonance imaging, gray matter density, dry eye disease, climacteric period

## Abstract

We aim to investigate potential morphological alterations of the brain in female climacteric patients with dry eye (DE) and their relationship to behavioral performances. Twenty-five female patients with DE disease during the female climacteric period and 25 age and education-matched healthy controls (HCs) underwent magnetic resonance imaging. Imaging data were analyzed using voxel-based morphometry (VBM) to identify group differences in DE patients and HCs. Compared with HCs, patients with DE during the female climacteric period had significantly decreased VBM in the Putamen_L, Thalamus_R, Precuneus_L, Frontal_Sup_R, Cingulum_Mid_L, and Frontal_Mid_L. There was increased VBM in the Temporal_Pole_Sup_R, Precentral_R and Insula_L. Receiver operating characteristic curve analysis indicated that the VBM method has clear potential for diagnosis of DE patients during the climacteric period. Correlation analysis found a negative correlation between the VBM values of the Putamen_L and the anxiety score (AS) and depression score (DS), a positive correlation was found between VBM values of the Temporal_Pole_Sup_R and AS. Moreover, VBM values in the Cingulum_Mid_L were positively correlated with AS and DS. These results revealed abnormal spontaneous activity in the brain regions of patients with DE during the climacteric period, which may indicate underlying pathological mechanisms. These results may help to advance clinical treatments.

## Introduction

Dry eye (DE) is a term used for a variety of diseases with eye surface tissue damage caused by an abnormal quantity or quality of tears or tear film instability caused by dynamic abnormalities. Eye discomfort is the main complaint and, in some severe cases, visual impairment may occur. Generally, DE can be divided into two types: insufficient tear production and excessive tear evaporation. The former is caused by lacrimal gland dysfunction, which has been termed aqueous tear deficiency (ATD). The latter mainly refers to meibomian gland dysfunction (MGD). DE is more prevalent in women than men and the incidence increases with age (1, 2). According to an epidemiological study, 11.3% of people more than 50 years old were diagnosed with DE, and that percentage increased to 22.8% in women more than 75 years old (1). Since DE not only causes visual impairment, which directly affects daily living, it also brings sociopsychological problems that reduce the quality of life. The increased need for therapy and reduced incomes due to decreasing productivity makes DE a cause of economic burdens (1–5).

The climacteric period, also known as menopause, refers to a period of time that correlates with the age-related decline in ovarian hormone production, usually starting at the ages of 49 to 52 years. Although statements about the climacteric period have been made in the medical literature for centuries, a scientific approach to understanding the climacteric years is a young branch of medicine (6). As mentioned above, DE is more prevalent in females, especially at the age of menopause. This is mainly because of hormonal disorders. Decreased hormonal levels may reduce production of tear film components (7). Several studies have suggested that hormone replacement therapy may have benefits in DE patients during the female climacteric period (8, 9). AlAwlaqi and Hammadeh showed an increased risk of DE symptoms in women taking hormone replacement therapy (10). Thus, the specific mechanism of hormonal disorders and DE remains unclear.

In recent years, research on the clinical diagnosis, treatment, and pathological development of ophthalmic diseases has increasingly proliferated with medical imaging technology. Voxel-based morphometry (VBM), as a new method of magnetic resonance imaging (MRI) analysis, can be used to quantitatively analyze alterations in the density and volume of white and gray matter using each individual voxel in magnetic resonance images, thus reflecting the anatomical structure differences in corresponding regions (11). This may provide an entirely new way to explore neuropathological changes with eye diseases. The VBM technique has already been used widely in many ophthalmic diseases, such as retinal detachment, monocular blindness, concomitant strabismus, optic neuritis, and acute eye pain, as well as other neurogenic diseases (Table 1). In our study, we aimed to determine whether structural changes exist in certain brain regions of female patients with DE during the climacteric period compared to healthy controls (HCs).

**Table 1:** VBM method applied in ophthalmologic and neurogenic diseases.

	**Author**	**Year**	**Disease**
**VBM method applied in ophthalmologic and neurogenic disease**			
	Li et al. (12)	2020	retinal detachment
Ophthalmologic Disease	Shi et al. (13)	2019	monocular blindness
	Ouyang et al. (14)	2017	comitant strabismus
	Huang et al. (11)	2016	optic neuritis
	Lan et al. (15)	2019	acute eye pain
Neurogenic Disease	González-Ortiz et al. (16)	2021	epilepsy
	Ting et al. (17)	2015	Alzheimer disease
	Ågren et al. (18)	2021	essential tremor
	Xuan et al. (19)	2019	Parkinson disease

## Materials and Methods

### Subjects

Twenty-five female patients with DE during the female climacteric period were recruited. Inclusive criteria were as follows: 1) primary DE diagnosed by an experienced ophthalmologist; 2) no menstruation for at least 12 months and met the criteria of climacteric period judged by the gynecologist; and 3) not treated with any drugs or had stopped treatment for at least 2 weeks before recruitment. Exclusive criteria were as follows: 1) conjunctival scarring, atresia, or complete atrophy of the lacrimal gland and accessory lacrimal glands; 2) other obvious abnormalities of the conjunctiva, cornea, or iris; 3) pregnant or nursing women; 4) history of mental health disorders, diabetes, cerebral infarction, or cardiovascular diseases; 5) history of addictions (alcohol and/or drugs); 6) abnormality of the brain parenchyma as shown by MRI; and 7) contraindication for MRI scanning.

Twenty-five female HCs were also enrolled. Their average age and educational level were similar to the participants in the DE group. All HCs met the following criteria: 1) best-corrected visual acuity >1.0; 2) no organic ocular diseases; 3) no menstruation for at least 12 months and met the criteria of climacteric period judged by the gynecologist; 4) no abnormalities of the brain parenchyma or visual pathway on head MRI scans; 5) no drug or alcohol addiction; 6) no mental illness or cerebral diseases, and 7) no contraindications for MRI.

The study was authorized by the Ethics Committee of the First Affiliated Hospital of Nanchang University. All participants were offered an explanation of the standard operation procedure (SOP) for MRI scans and signed the consent form. All the protocols in the study followed the Declaration of Helsinki and conformed to the principles of medical ethics.

#### Administration of the Hospital Anxiety and Depression Scale (HADS)

The HADS was used to quantitatively assess anxiety and depression (20). For participants who were illiterate or were unable to read due to visual impairment, an investigator read the questionnaire aloud. For all participants, an investigator verbally explained the purpose of the questionnaire and its confidential nature. We calculated the anxiety score (AS) and depression score (DS) separately.

#### Structural MRI Parameters

A 3T MR scanner (Siemens, Munich, Germany) with a 12-channel head coil was used to perform MRI scanning. We acquired high-resolution T1-weighted functional images using a magnetization-prepared rapid gradient echo (MP-RAGE) sequence covering the entire brain. The detailed parameters were as follows: 176 slices with section thickness of 1.0 mm; echo time = 2.26 ms; repetition time = 1,900 ms; and field of view = 215 × 230 mm. The scanning procedure was carried out for all participants by the same experienced radiologist and no participants were excluded.

#### Image Processing

For data acquisition and processing, MRIcron (http://www.nitrc.org/projects/mricron) was employed to classify functional data and eliminate incomplete data. The structural images were processed with a voxel-based morphometry toolbox (VBM8, dbm.neuro.uni-jena.de/vbm8) on a statistical parameter map (SPM8; http://www.fil.ion.ucl.ac.uk). All procedures were carried out using MATLAB7.9.0 software (The MathWorks, Natick, MA). Based on VBM8, individual brains were divided into gray matter (GM), white matter (WM), and cerebrospinal fluid (CSF). More details were presented in a previous study (13).

#### Processing of Data

Using the SPM8 toolkit, we applied general linear model analysis (GLM) to investigate the differences in GM and WM between DE patients and HCs after controlling for age. *P* < 0.05 was considered statistically significant, and Gaussian random field (GRF) theory correction was used for multiple comparisons (minimum z >2.3). To generate a colored map, significant voxels were superimposed on the normalization of three-dimensional magnetization, fast acquisition gradient echo sequences (3DT1WIs). Area analysis of gray matter changes was performed at the voxel threshold setting as 20 neighboring voxels.

Using REST1.8 software (www.resting-fmri.Sourceforge.net), distinct brain regions were defined as regions of interest (ROIs). We then used receiver operating characteristic (ROC) curves for each ROI to explore the relationship between mean outcomes and clinical features in different regions of the brain.

#### Statistical Analysis

We used two-sample *t*-tests to compare VBM values at the ROIs between the DE and HCs groups with age as covariates to control for these factors. ROC curves were also used to assess sensitivity of the discrimination between mean VBM values in the two groups. In addition, Pearson's correlation analysis was applied to VBM and HADS scores. SPSS version 19.0 statistical software for Windows (SPSS, IBM Corp, USA) was used to analyze the data, and GraphPad Prism 8.0 software (the GraphPad Software, Inc. La Jolla, CA) was applied for figure generation. ^*^ meant the *P*-value was <0.05 and was considered statistically significant in all cases.

## Results

### Demographics and Visual Measurements

The best-corrected visual acuity was significantly decreased in both eyes of DE patients, compared with HCs (*p* = 0.034, right eyes; *p* = 0.032, left eyes.) No significant differences were found in weight (*p* = 0.892) or age (*p* = 0.854) between the DE patients and HCs (Table 2).

**Table 2:** Clinical characteristics of patients in the DE and HC groups.

**Characteristics**	**DE**	**HCs**	***t*-value**	***P*-values**
Age(years)	56.76 ± 10.16	57.12 ± 11.68	−0.319	0.854
Weight(kg)	58.64 ± 7.91	59.12 ± 6.75	−0.462	0.892
Handedness(left/right)	0/25	0/25	NA	NA
Duration of DE (mons)	34.32 ± 13.11	NA	NA	NA
Best-corrected VA, right	0.75 ± 0.33*	1.17 ± 0.69	−3.142	0.034
Best-corrected VA, left	0.67 ± 0.24*	1.21 ± 0.46	−2.169	0.032

Independent t-tests comparing the two groups (*P <0.05) showed statistically significant differences.

*DE, dry eye; HCs, healthy controls; NA, not applicable; VA, visual acuity*.

#### VBM Values and Brain Regions

**Figure 1:**
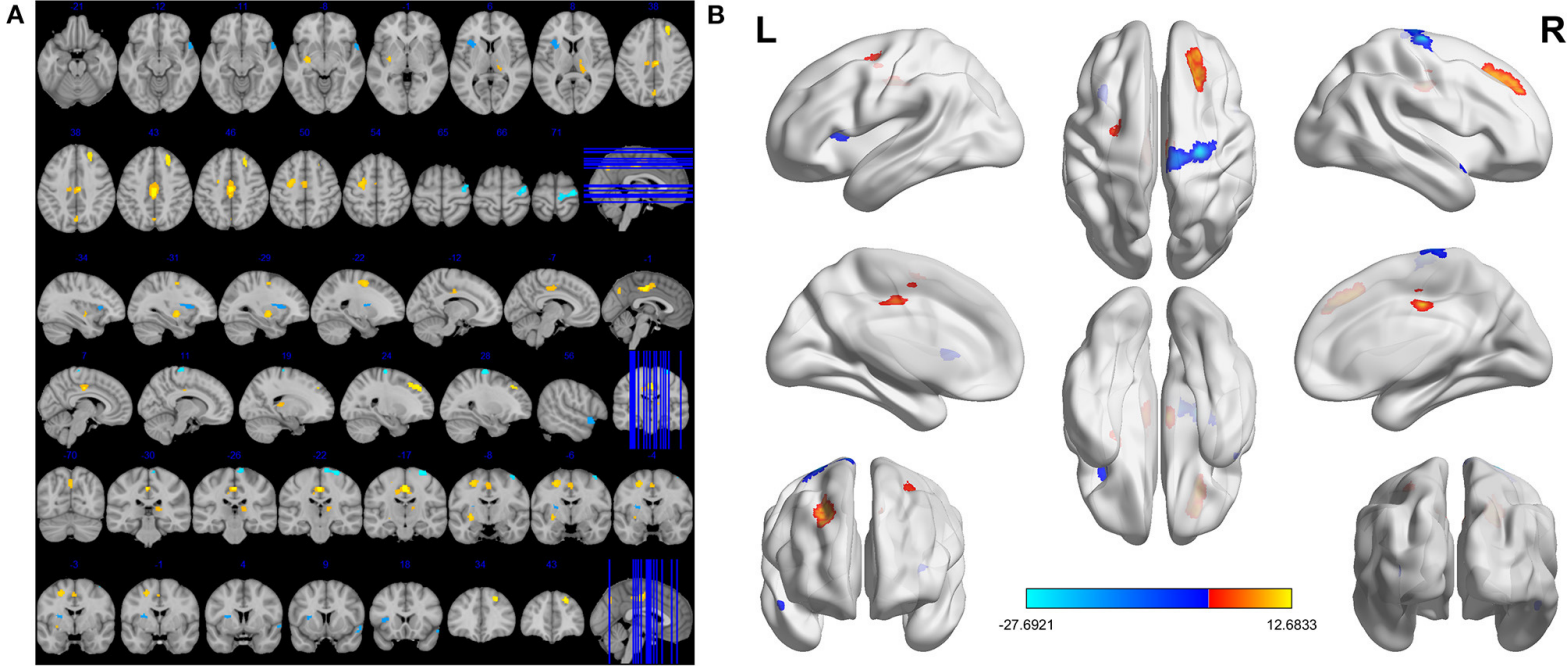
Significant differences in VBM values between DE patients and HCs. The brain regions with different VBM values were the Precuneus_L, Putamen_L, Frontal_Sup_R, Frontal_Mid_L, Thalamus_R, Cingulum_Mid_L, Insula_L, Precentral_R, Temporal_Pole_Sup_R **(A)**. The read areas denote higher fALFF brain regions and blue areas denote lower fALFF brain regions **(B)**.

**Figure 2:**
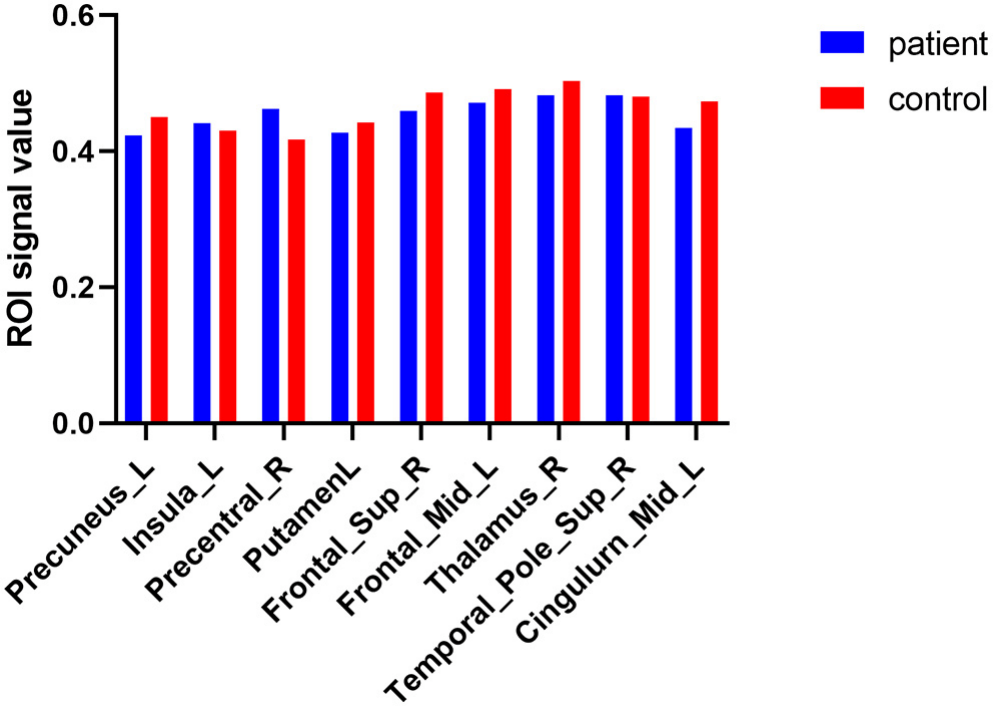
The average VBM values of DE patients and HCs. Compared with HCs, DE patients had significantly decreased VBM values in the left putamen, right thalamus, left precuneus, right superior frontal lobe, left middle cingulum and left middle frontal lobe; increased VBM in the right superior temporal_pole, right precentral area, and left insula lobe.

Compared to HCs, DE patients had significantly increased VBM in the Temporal_Pole_Sup_R (*t* = −4.1164), Precentral_R (*t* = −27.6921) and Insula_L (*t* = −4.6003), decreased VBM values in the Putamen_L (*t* = 7.5285), Thalamus_R (*t* = 5.4554), Precuneus_L (*t* = 6.5564), Frontal_Sup_R (*t* = 9.7716), Cingulum_Mid_L (*t* = 12.6833) and Frontal_Mid_L (t = 7.7449; Figures 1, 2, Table 3).

**Table 3:** Brain areas with significantly different VBM values between the groups.

**Brain areas (DE vs HCs)**	**BA**	**MNI coordinates**			**Voxels number**	**T-value**
		**X**	**Y**	**Z**		
Temporal_Pole_Sup_R	38	54	16	−12	98	−4.1164
Putamen_L	/	−30	−10	−4	130	7.5285
Insula_L	13	−26	−2	16	83	−4.6003
Thalamus_R	/	16	−22	8	122	5.4554
Precuneus_L	7	0	−70	38	108	6.5564
Frontal_Sup_R	8	24	40	42	194	9.7716
Cingulum_Mid_L	24	0	−18	46	513	12.6833
Frontal_Mid_L	6	−26	−8	52	209	7.7449
Precentral_R	6	26	−20	70	265	−27.6921

*The statistical threshold was set at voxels with p <0.01 for multiple comparisons using Gaussian random field corrections.*

*MNI, Montreal Neurological Institute; BA, Brodmann's area*.

#### 3.3. ROC Curve Analysis

**Figure 3:**
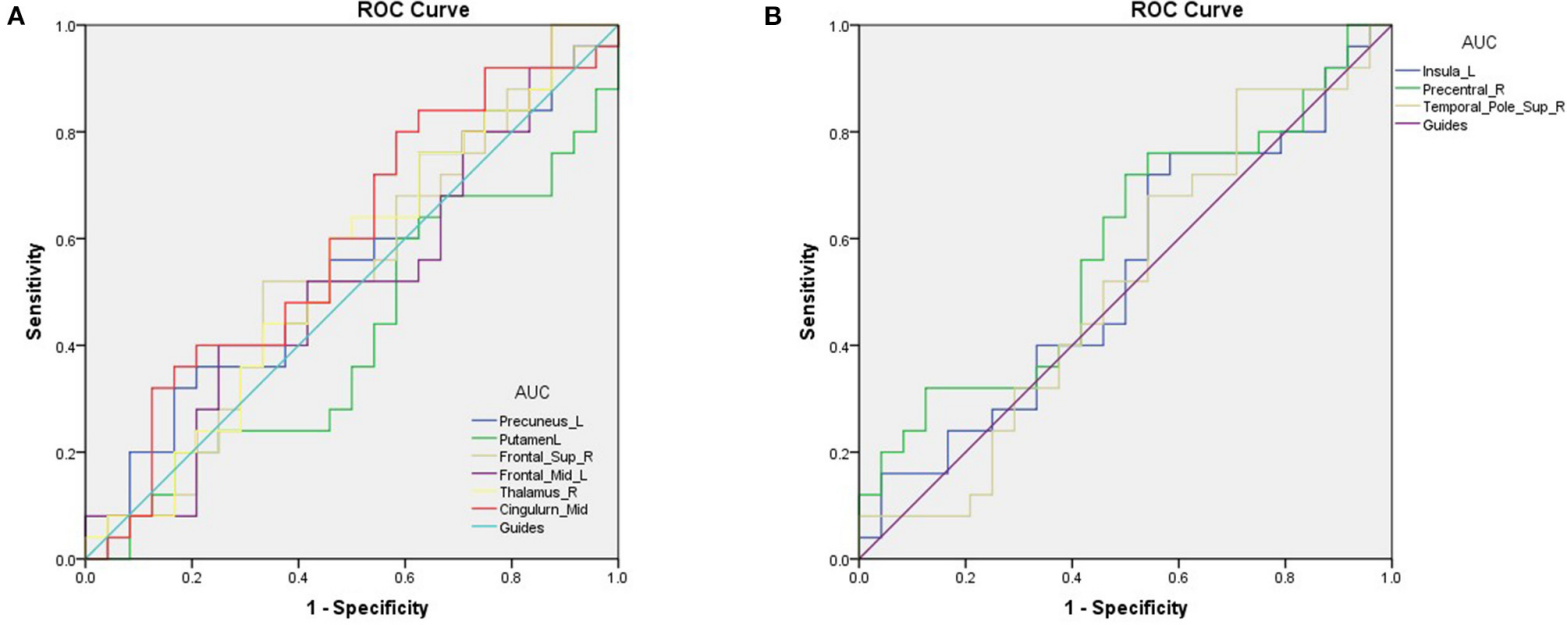
ROC curve analysis of the mean VBM values for altered brain regions. **(A)** The areas under the ROC curve were 0.548 for Precuneus_L (*p* = 0.562; 95% CI: 0.385–0.711); Putamen_L 0.415, (*p* = 0.308; 95% CI: 0.253–0.577); Frontal_Sup_R 0.525, (*p* = 0.764; 95% CI: 0.360–0.690); Frontal_Mid_L 0.518, (*p* = 0.826; 95% CI: 0.353–0.683); Thalamus_R 0.545, (*p* = 0.589; 95% CI: 0.381–0.709); Cingulum_Mid_L 0.590, (*p* = 0.280; 95% CI: 0.428–0.752). **(B)** The areas under the ROC curve were 0.532 for the Insula_L (*p* = 0.704; 95% CI: 0.367–0.696); 0.585 for the Precentral_R (*p* = 0.308; 95% CI: 0.423–0.747) and 0.517 for the Temporal_Pole_Sup_R (*p* = 0.841; 95% CI: 0.351–0.682). AUC, area under the curve; ROC, receiver operating characteristic.

The area under the ROC curves were 0.548 for the Precuneus_L (*p* = 0.562; 95% CI: 0.385–0.711); 0.415 for the Putamen_L (*p* = 0.308; 95% CI: 0.253–0.577); 0.525 for the Frontal_Sup_R (*p* = 0.764; 95% CI: 0.360–0.690); 0.518 for the Frontal_Mid_L (*p* = 0.826; 95% CI: 0.353–0.683); 0.545 for the Thalamus_R (*p* = 0.589; 95% CI: 0.381–0.709); and 0.590 for the Cingulum_Mid_L (*p* = 0.280; 95% CI: 0.428–0.752; Figure 3A). The area under the ROC curves were 0.532 for the Insula_L (*p* = 0.704; 95% CI: 0.367–0.696); 0.585 for the Precentral_R (*p* = 0.308; 95% CI: 0.423–0.747); and 0.517 for the Temporal_Pole_Sup_R (*p* = 0.841; 95% CI: 0.351–0.682; Figure 3B).

#### Correlation Analysis of the VBM Values and Clinical Measurements in DE Patients

**Figure 4:**
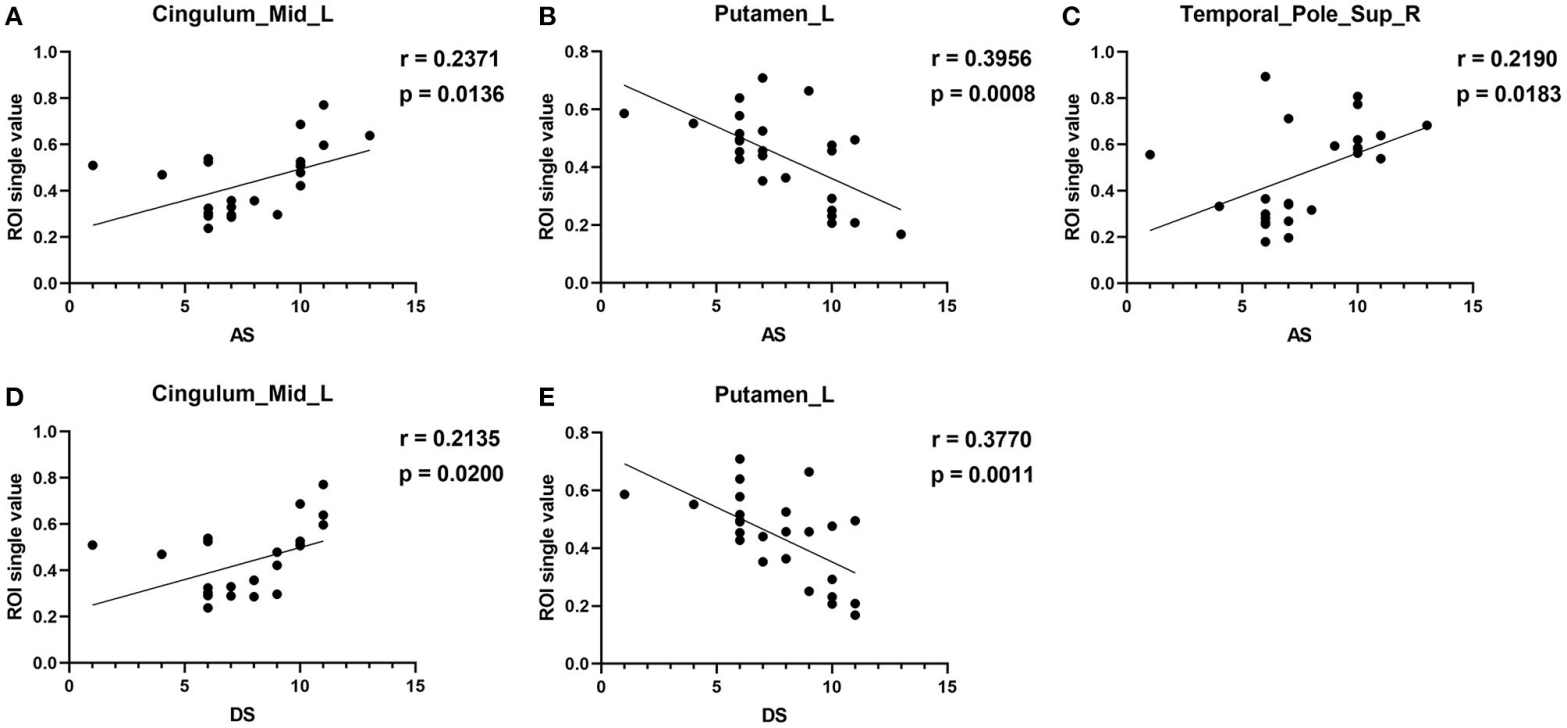
Correlation between the VBM values of the brain regions and HADS. In the DE group, the VBM values of the Cingulum_Mid_L showed a positive correlation with the AS [r = −0.2371, *p* = 0.0136, **(A)**] and DS [r = 0.2135, *p* = 0.0200, **(D)**]. Mean VBM values in the Putamen_L of DE patients were negatively correlated with the AS [r = 0.3956, *p* = 0.0008, **(B)**] and DS [r = 0.3770, *p* = 0.0011, **(E)**]. Mean VBM values in the Temporal_Pole_Sup_R of DE patients were positively correlated with AS **(C)** [r = 0.2190, *p* = 0.0183].

Mean VBM values in the Cingulum_Mid_L of DE patients were positively correlated with the AS (r = 0.2371, *p* = 0.0136) and DS (r = 0.2135, *p* = 0.0200). The mean VBM values in the Putamen_L of DE patients were negatively correlated with the AS (r = 0.3956, *p* = 0.0008) and DS (r = 0.3770, *p* = 0.0011). The mean VBM values in the Temporal_Pole_Sup_R of DE patients were positively correlated with the AS (r = 0.2190, *p* = 0.0183; Figure 4). No correlation was found between the duration of the disease and VBM values in any of the brain regions.

## Discussion

**Figure 5:**
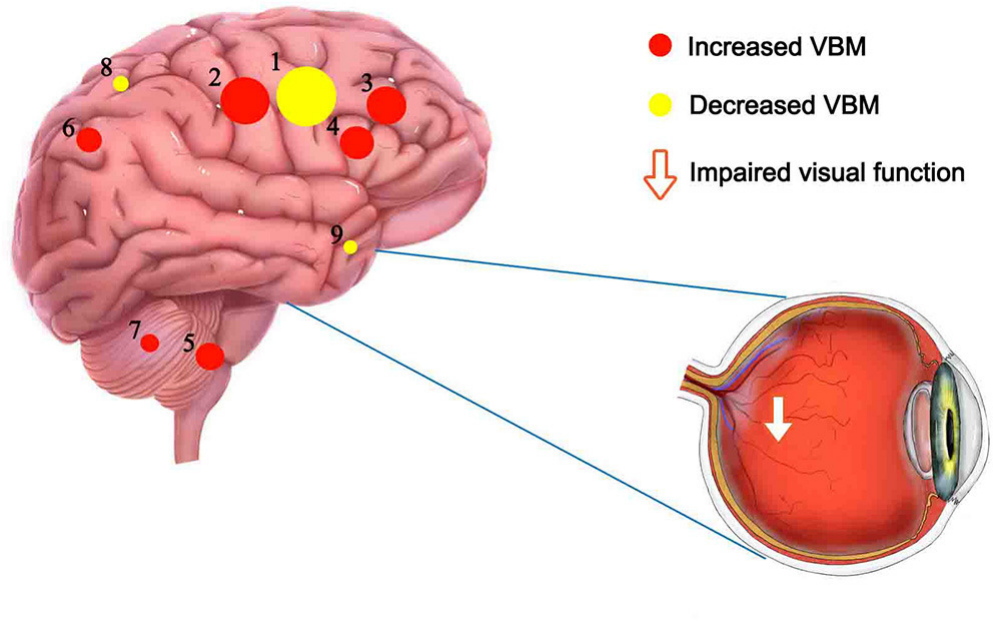
Mean VBM values of altered brain regions. Compared to HC subjects, the VBM values of the following regions were decreased to various extents: 6- Precuneus_L (t = 6.5564), 5- Putamen_L (t = 7.5285), 3- Frontal_Sup_R (t = 9.7716), 4- Frontal_Mid_L (t = 7.7449), 7-Thalamus_R (t = 5.4554), 2- Cingulum_Mid_L (t = 12.6833). Compared with the HCs, the VBM values of the following regions were increased to various extents: 8-Insula_L (t = −4.6003), 1- Precentral_R (t = −27.6921), 9- Temporal_Pole_Sup_R (t = −4.1164). HCs, healthy controls; BA, Brodmann's area.

This is the first study that investigated changes in brain regions in DE patients during the female climacteric period using a VBM approach. In our study, compared to HCs, significantly decreased VBM values was found in the left putamen, right thalamus, left precuneus, right superior frontal lobe, left middle cingulum, and left middle frontal lobe. Meanwhile, increased VBM values were found in the right superior temporal pole, and right precentral and left insula (Figure 5). ROC curve analysis indicated the potential role of the VBM method for diagnosing DE patients during the climacteric period. Correlation analysis suggested a correlation between the VBM values of certain brain regions in DE patients and their HADS score, but no correlation was found between the duration of the disease and VBM values. Namely, the VBM values of the Putamen_L were negatively correlated with the AS and DS, while a positive correlation was found between VBM values in the Temporal_Pole_Sup_R and the AS. Moreover, VBM values in the Cingulum_Mid_L of DE patients were positively correlated with the AS and DS.

The precuneus is the posterior part of the medial parietal cortex (21). Recent functional imaging findings suggested that the precuneus may participate in visuo-spatial imagery and other highly integrated works (22). The precuneus may also be responsible for self-consciousness (23). In our study, lower VBM values of the Precuneus_L were found in DE patients compared to HCs, suggesting that visual impairment secondary to DE may be correlated with changes in the precuneus lobe.

The thalamus is located in the diencephalon and is comprised of four parts (24). It is a vital relay center for both sensory and motor mechanisms and has several important functions, including sensory and motor functions, sleeping, and emotions (22). Basilious summarized 32 studies and concluded that the prevalence of depression and anxiety are much higher in DE patients (25). In another study, DE patients showed sleep quality reduction (26). In addition, menopausal women with sexual dysfunction also reported a higher prevalence of depression and anxiety (27), as well as sleeping disorders (28). In the present study, the VBM values of Thalamus_R were obviously decreased in DE patients compared to HCs, which corresponded with emotional changes (depression and anxiety) and sleep disorders in DE patients during menopause.

The putamen is part of the lentiform nucleus, which constitutes part of the basal ganglia with other nuclei (29). The putamen is mainly responsible for learning and motor control, as well as cognitive function (30, 31). Kuru found putamen abnormalities in patients with various motor and cognitive dysfunctions (32). In our study, the VBM values of Putamen_L in DE patients were lower and negatively correlated with HADS scores, indicating that these patients may have cognitive disfunction or movement disorders.

The frontal lobe is located anteriorly to the central sulcus and superiorly to the lateral sulcus. The external part of the frontal lobe is divided into three large surfaces (33). In recent years, the middle frontal gyrus (MFG) has been suggested to be part of the language network (34). In addition, Carter's research indicated the role of MFG in stress and cognitive functions (35). The superior frontal gyrus (SFG) has been linked to depression, cognition, and attention (36). Chang and his colleagues found SFG abnormalities in patients with depression (37). Thus, dysfunction of the frontal lobe may result in emotional and cognitive problems. In fact, we found VBM values declined in the frontal lobe, which was in accordance with our previous study (38).

The temporal lobe is a complex region. It has diverse cortical functions. Abnormal activation of these brain regions may cause epilepsy, schizophrenia, and amnesia (39). The insular cortex is buried inside the lateral sulcus of the brain (40). It can be divided into four functional parts with motor, olfactogustatory, socioemotional, and cognitive functions (41). The precentral gyrus is located on the lateral surface of the frontal lobe. It is involved in controlling voluntary motor movement (42). Another study found a correlation between decreased FA values in the precentral gyrus and age processing (43). Brain region alternation and its potential impact was summarized in Table 4. In this study, we demonstrated increased VBM values in the above brain regions in DE patients during menopause. Abnormal activity in these brain regions may be correlated with some excessive symptoms of DE patients, such as anxiety. Given that other functionally-similar brain regions showed contradictory low VBM values, compensation mechanisms may also exist in these brain regions.

**Table 4:** Brain region alternations and their potential impact.

**Brain regions**	**Experimental result**	**Brain function**	**Anticipated results**
Temporal_Pole_Sup_R	DE > HC	electro physiologic networks, language	Schizophrenia, epilepsy, anterograde amnesia
Putamen_L	DE < HC	learning and motor control	Eye movements, learning problems
Insula_L	DE > HC	socioemotional, sensorimotor, processing, cognitive functions	Depression, cognitive activity disorder
Thalamus_R	DE < HC	sensory and motor function, attention, memory, speech, and emotion	Tremor, sleep disorder, depression, etc.
Precuneus_L	DE < HC	visuo-spatial imagery, episodic memory retrieval, self-consciousness	Visual impairment, amnesia
Frontal_Sup_R	DE < HC	Cognitive, emotional, pain, and behavioral management	Irritability, mood swings, depression, etc.
Cingulum_Mid_L	DE < HC	Executive functions, attention and memory	depression, pain, and anxiety
Frontal_Mid_L	DE < HC	Key parts of word processin	Cognitive activity disorder
Precentral_R	DE > HC	voluntary motor movement, aging process	Eye movements, climacteric period

A variety of systemic comorbidities has been reported in patients with DE and other ocular diseases, such as problems with memory, depression, cognition, and sleep (44). More severe organic symptoms, sensory and sensorimotor neuropathies, and movement disorders have also been reported (45). We believe that these symptoms may be related to changes in the VBM values of the brain regions mentioned above. Our research demonstrated that the clinical symptoms of DE patients were indeed associated with brain dysfunctions. Thus, we offer two assumptions: 1) DE causes emotional instability, which aggravates endocrine disorders and makes menopausal symptoms more obvious; and 2) abnormal endocrine function in menopause leads to poor lacrimal gland function, meibomian gland dysfunction, and then finally develops to DE.

There are some limitations in our study. First, DE can be divided into many different types, and our sample size was not large enough to subdivide patients into subgroups. Larger sample MRI studies may help increase understanding of the explicit changes in brain activity and DE at different ages or in different categories. Second, both DE and the climacteric period may cause psychological problems, thus impacting brain activity. Whether brain activity changes are secondary to DE, further aggravate symptoms of the climacteric period, or that the climacteric period caused brain activity changes and then contributes to DE remains unknown. In the future, larger more in-depth studies may help to address this question.

## Conclusions

In conclusion, our study found significant abnormalities in certain brain regions in patients with DE during the climacteric period. ROC analyses indicated high accuracy of the VBM method for diagnosing DE patients. In the future, further investigations are needed to form a comprehensive understanding of the neuropathological mechanisms of DE.

## Data Availability Statement

The raw data supporting the conclusions of this article will be made available by the authors, without undue reservation.

## Ethics Statement

The studies involving human participants were reviewed and approved by the committee for medical ethics of the First Affiliated Hospital of Nanchang University. The patients/participants provided their written informed consent to participate in this study.

## Author Contributions

M-YH, YS, and C-GP designed the current study. MK, M-YH, and L-JZ recruited healthy controls. MK, Y-CP, and L-JZ performed MRI scanning. LY, Q-mG, and Q-yL collected and analyzed the data. M-YH wrote the manuscript. All the authors read and approved the final manuscript.

## Funding

This research was supported by Key Research Foundation of Jiangxi Province (Nos. 20203BBG73059 and 20181BBG70004), Excellent Talents Development Project of Jiangxi Province (20192BCBL23020), Health Development Planning Commission Science Foundation of Jiangxi Province (20175115), and Education Department Youth Scientific Research Foundation (GJJ160122).

## Conflict of Interest

The authors declare that the research was conducted in the absence of any commercial or financial relationships that could be construed as a potential conflict of interest.

## Publisher's Note

All claims expressed in this article are solely those of the authors and do not necessarily represent those of their affiliated organizations, or those of the publisher, the editors and the reviewers. Any product that may be evaluated in this article, or claim that may be made by its manufacturer, is not guaranteed or endorsed by the publisher.
